# Activin signaling is an essential component of the TGF-β induced pro-metastatic phenotype in colorectal cancer

**DOI:** 10.1038/s41598-017-05907-8

**Published:** 2017-07-17

**Authors:** Jonas J. Staudacher, Jessica Bauer, Arundhati Jana, Jun Tian, Timothy Carroll, Georgina Mancinelli, Özkan Özden, Nancy Krett, Grace Guzman, David Kerr, Paul Grippo, Barbara Jung

**Affiliations:** 1 Department of Medicine, Division of Gastroenterology and Hepatology, University of Illinois at Chicago, Chicago, IL 60612 USA; 2 Department of Pathology, University of Illinois at Chicago, Chicago, IL 60612 USA; 3 Nuffield Division of Clinical Laboratory Sciences, University of Oxford, Oxford, UK

## Abstract

Advanced colorectal cancer (CRC) remains a critical health care challenge worldwide. Various TGF-β superfamily members are important in colorectal cancer metastasis, but their signaling effects and predictive value have only been assessed in isolation. Here, we examine cross-regulation and combined functions of the two most prominent TGF-β superfamily members activin and TGF-β in advanced colorectal cancer. In two clinical cohorts we observed by immune-based assay that combined serum and tissue activin and TGF-β ligand levels predicts outcome in CRC patients and is superior to single ligand assessment. While TGF-β growth suppression is independent of activin, TGF-β treatment leads to increased activin secretion in colon cancer cells and TGF-β induced cellular migration is dependent on activin, indicating pathway cross-regulation and functional interaction *in vitro*. mRNA expression of activin and TGF-β pathway members were queried *in silico* using the TCGA data set. Coordinated ligand and receptor expression is common in solid tumors for activin and TGF-β pathway members. In conclusion, activin and TGF-β are strongly connected signaling pathways that are important in advanced CRC. Assessing activin and TGF-β signaling as a unit yields important insights applicable to future diagnostic and therapeutic interventions.

## Introduction

Colorectal cancer (CRC) is the third most common cancer worldwide resulting in approximately 50,000 deaths per year in the US alone^1^. While the overall incidence of CRC is decreasing likely due to increased screening resulting in prevention or early detection^2^, the prognosis of late stage disease remains poor. Risk stratification for metastatic disease continues to be challenging. In addition there is no consensus on management for suspected early metastatic disease and use of adjuvant or post-surgery chemotherapy in stage 2 CRC remains controversial^3^.

Given the relatively low recurrence rates in stage 2 CRCs^4^, the risk benefit ratio does not favor treating all patients for suspected metastasis. However, there is a small but important subpopulation of stage 2 CRC patients that later present with metastases and likely would benefit from early systemic treatment^5^. Once CRC has metastasized distally, palliative and supportive approaches are the standard of care^6^ with a five year survival rate as low as 10%^1^. Therefore, both new treatment approaches and biomarkers for risk stratification for metastatic disease are needed in CRC.

TGF-β superfamily signaling is established as a crucial signaling family in CRC^7^, with a dominant growth suppressive function at early stages of tumorigenesis, yet parallel pro-metastatic effects at later time points^8^. The most involved superfamily members in sporadic CRC are TGF-β and activin, each having several ligand isoforms. In this study, we chose to focus on the most prominent isoforms of activin and TGF-β in CRC, activin A and TGFB1, respectively as they are the two cancer dominant isoforms.

After ligand binding to respective type II receptors, both signal through subsequent dimerization with and phosphorylation of their specific type I receptors^9^ (also called activin receptor like kinases or ALKs) to activate shared canonical and distinct non-canonical pathways^10^.

In the canonical pathway phosphorylation of SMAD 2 and/or 3, is followed by dimerization with SMAD4^11^ and SMAD2/3-SMAD4 complex translocation to the nucleus, where a substantial number of genes are either transcriptionally activated or repressed^12^. In contrast, a number of CRC oncogenic pathways, including but not limited to PI3K/AKT^13, 14^, MAPK/ERK^13, 15^, WNT^16^, and notch^17^, can be activated by TGF-β superfamily signaling independent of SMADs and are thus called non-canonical pathways.

We have previously reported that while activin and TGF-β share canonical signaling, in CRC they diverge in their non-canonical pathways with activin primarily signaling through PI3K/AKT while TGF-β activates MAP/ERK^13^. Numerous ligand isoforms, ligand promiscuity of some type I and type II receptors, and substantial crosstalk between canonical and non-canonical pathways contribute to a complex yet poorly understood system which allows context-specific and dynamic functions of TGF-β superfamily signaling^10^.

This is emphasized by the fact that despite a number of available compounds for TGF-β pathway inhibition^18^ and promising data in animal models^19–21^, to date no clinical benefit of targeting TGF-β signaling has been reported in solid tumors^22^. In *vivo* data showed that disruption of epithelial TGF-β receptor signaling may lead to a more invasive and aggressive CRC phenotype through unknown mechanisms^23, 24^ likely involving stromal signaling. Recent data from our group implies that TGF-β inhibition through neutralization of TGF-β ligand may be detrimental in a subset of CRCs^25^.

Given activin and TGF-β’s overlap in their canonical pathway and divergence in their downstream oncogenic signaling as well as the frequent co-occurrence of mutations in their respective receptors^26–28^, we here focus on understanding the mechanisms of activin and TGF-β crosstalk in metastatic signaling in CRC.

## Results

### An elevated combined activin/TGF-β ligand expression score is predictive of a worse prognosis in patients with CRC

Because of the overlap of the canonical and divergence of non-canonical activin and TGF-β functions, we hypothesized that both pathways need to be assessed simultaneously to comprehensively predict TGF-β superfamily effects. To test our hypothesis, we determined activin and TGF-β serum ligand levels in a subset of 120 stage II patients (60 with and 60 without recurrence) from the Quasar trial^29^ and correlated both single ligands and a combined ligand score with clinical outcome.

**Figure 1:**
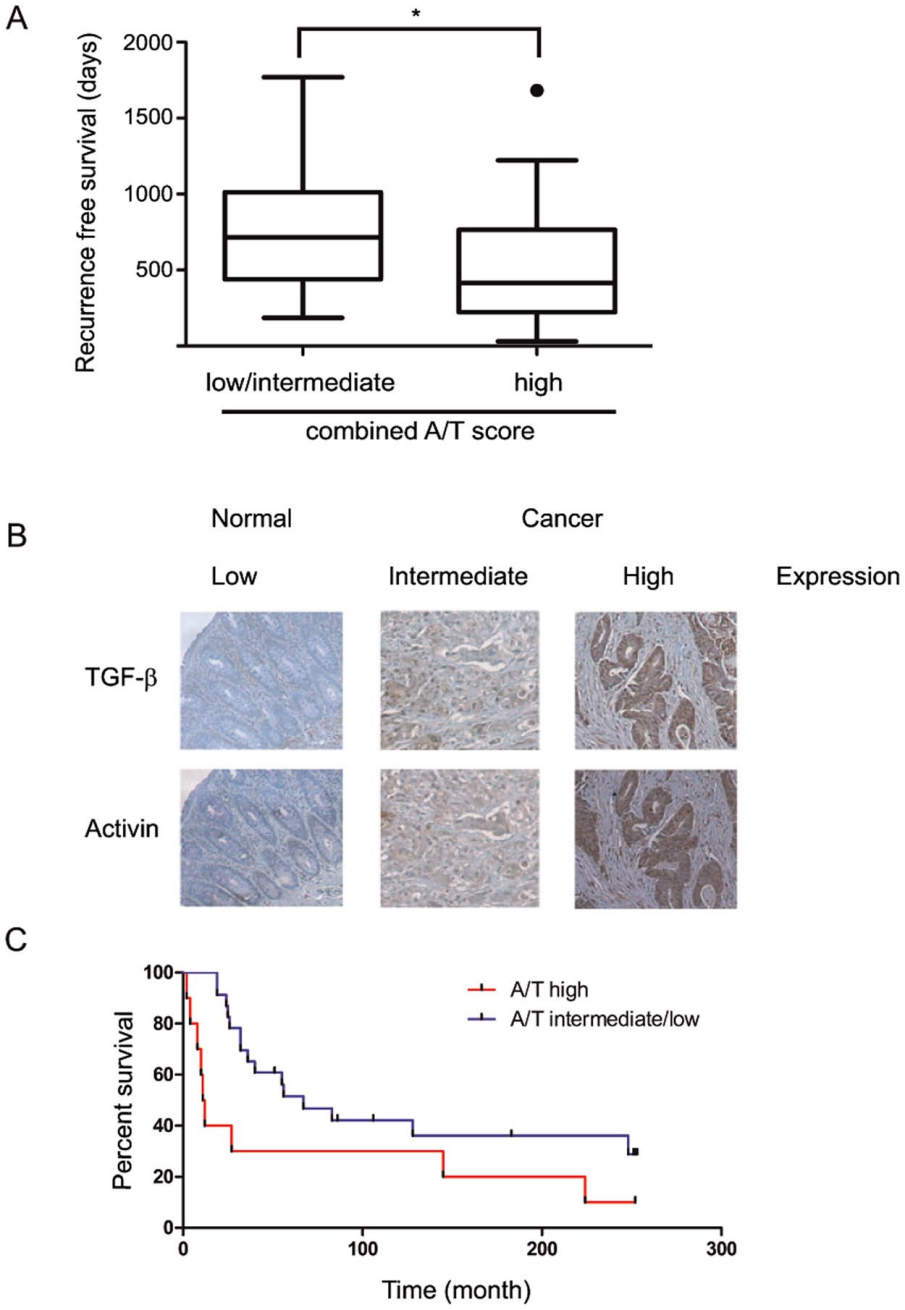
Combined activin and TGF-β ligand scores are strongly predictive of a worse prognosis in colorectal cancer. (**A**) Serum ligand levels are predictive of shorter recurrence-free survival. Sixty stage 2 CRC patients with recurrence were stratified by activin and TGF-b serum ligand expression and with median recurrence-free survival shown as box plots. The combined activin/ TGF-β score (A/T) is defined in the Materials and Methods. Low/intermediate is defined as a score of 4 or less and high is defined as a score of 5 or higher. (**B**) Tumoral activin and TGF-β expression are strongly correlated. Representative sections of immunohistochemical staining of TGF-β or activin ligand in a CRC TMA with examples of low, intermediate and high staining. Matched colon cancer sections of adjacent slides are shown to demonstrate correlation of activin and TGF-β expression. (**C**) Tumoral activin and TGF-β ligand are predictive of shorter overall survival. Kaplan Meier curve of patient survival in either activin/ TGF-β high (A/T) patients or A/T low as defined in the Materials and Methods.

We found that ligand serum levels of activin and TGF-β significantly correlated with each other (r = 0.435, p < 0.05). Neither serum activin nor TGF-β alone significantly correlated with time to recurrence. However, a high combined activin/TGF-β score (see methods) was predictive of decreased recurrence-free survival compared to patients with low or intermediate activin/TGF-β serum levels (median recurrence free survival 415 days versus 715 days, p < 0.05, Fig. 1A), supporting our hypothesis of a combined activin/TGF-β system with pro-metastatic action.

To control for potential non-tumoral factors that may influence activin and TGF-β serum levels, we next determined tumor expression levels of the ligands. For this, we stained adjacent slides of a commercial TMA with 40 CRC, 10 CRC metastasis and 10 control samples^30^ for activin and TGF-β and correlated expression with each other as well as outcome (example staining, Fig. 1B). Activin and TGF-β tissue ligand expression scores correlated with each other (r = 0.604, p < 0.01). When we correlated activin and TGF-β expression with survival, activin and TGF-β combined, but not activin or TGF-β tissue ligand expression alone, was associated with statistically significant shorter survival (median overall survival 11.5 months versus 67 months, p < 0.05, Fig. 1C). Importantly, this correlation was not solely due to higher expression levels at higher stages (mean combined score stage III-IV 2.19 versus stage I-II 2.23 p = n.s.), indicating that combined tumor activin and TGF-β ligand expression provides additional information to the TNM staging classification^31^ regarding advanced disease.

### TGF-β leads to enhanced epithelial and stromal activin secretion and resulting migration is dependent on activin

Given the strong correlation and predictive power of activin and TGF-β ligand expression in our human cohorts, we next explored the impact of TGF-β on activin secretion. As the tumor microenvironment appears essential to TGF-β superfamily signaling, we also assessed the contribution of fibroblasts to activin ligand secretion using a co-culture approach.

**Figure 2:**
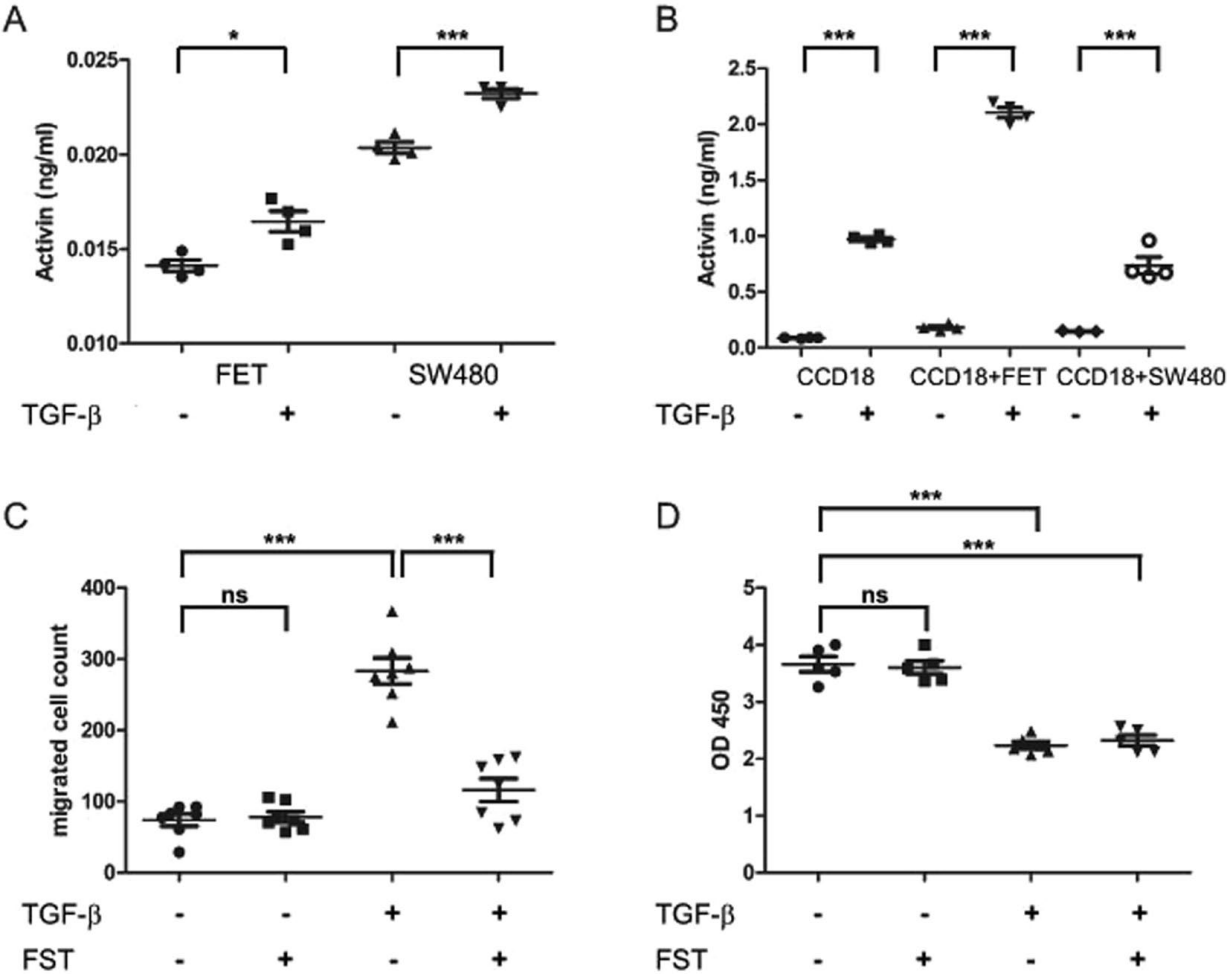
TGF-β’s pro-metastatic function is activin dependent while its anti-proliferative function is independent of activin. TGF-β increases activin secretion in both epithelial (**A**) and stromal (**B**) cells. (**A**) Activin ligand from respective CRC cell culture supernatants as measured by ELISA with and without treatment with 10 ng/ml of TGF-β. (**B**) Activin ligand from CRC cells with or without co-culture with CCD18 stromal cells. Cells were treated with either vehicle control (PBS) or 10 ng/ml TGF-β. (**C**) TGF-β pro-migratory effects are activin dependent. Transwell migration assay of FET colon cancer cells treated with either the activin inhibitor follistatin (FST, 100 ng/ml) or TGF-β (10 ng/ml) or both as indicated. Cells were imaged and counted as described in Materials and Methods. D) TGF-β induced anti-proliferative effects are activin independent. Metabolic activity as an approximation of cell proliferation of FET CRC cells treated with either follistatin (FST, 100 ng/ml) or TGF-β (10 ng/ml) or both as indicated was measured by wst-8-based cell counting assay and reported as OD450. All experiments shown were reproduced at least twice with at least a total n = 5.

Stromal CCD18 and epithelial CRC FET cells were either grown separately, or in co-culture using a trans-well approach and activin ligand expression analyzed following TGF-β stimulation. After TGF-β treatment, activin secretion was increased both in SMAD4 wild-type FET and SMAD4 null SW480 colon cancer cells (Fig. 2A). Interestingly, levels of activin secretion both at baseline and after TGF-β treatment were substantially higher either in stromal CCD18 cells alone or in co-cultures of stromal with epithelial cells, indicating that the stroma is a significant source of secreted activin (Fig. 2B).

As TGF-β showed a substantial impact on activin secretion, we next explored its effects on pro-metastatic functions. For this, we examined cell motility measured by transwell migration. As reported previously^13, 32^ and confirmed in Supplementary Figure S1, both activin and TGF-β individually increase the number of FET colon cancer cells which migrate through transwells. To delineate how much of the TGF-β migratory effect is dependent on activin, here we inhibited activin signaling using its specific antagonist follistatin (FST), which does not bind TGF-β but neutralizes activin with high affinity by acting as a ligand trap^33, 34^. We found that the pro-migratory effect of TGF-β was abrogated in the presence of follistatin, indicating that the TGF-β induced pro-metastatic function is dependent on activin signaling. Consistent with this, follistatin alone did not change migration (Fig. 2C). Similar, albeit more modest effects were observed in SMAD4 mutated SW480 colon cancer cells (Supplementary Figure S2).

To investigate the impact of activin inhibition on the anti-proliferative actions of TGF-β, we next measured metabolic activity following TGF-β treatment in the presence and absence of follistatin. Interestingly, in the presence or absence of TGF-β, follistatin had no impact on proliferation in TGF-β superfamily signaling wild-type FET colon cancer cells (Fig. 2D), confirming that TGF-β induced activin pathway activation selectively affects colon cancer cell migration and not TGF-β- induced growth suppression.

### Activin and TGF-β pathway member expression correlate on receptor and ligand level in patients with CRC

To further test our hypothesis that activin and TGF-β expression and signaling are closely connected in colorectal cancer and to further support data obtained in our clinical cohorts, we performed an *in silico* analysis using The Cancer Genome Atlas (TCGA) colorectal dataset^35^. This is a publicly available and NIH curated *in silico* data base providing high quality mRNA-seq data from solid tumor tissues compared to paired normal tissue.

**Figure 3:**
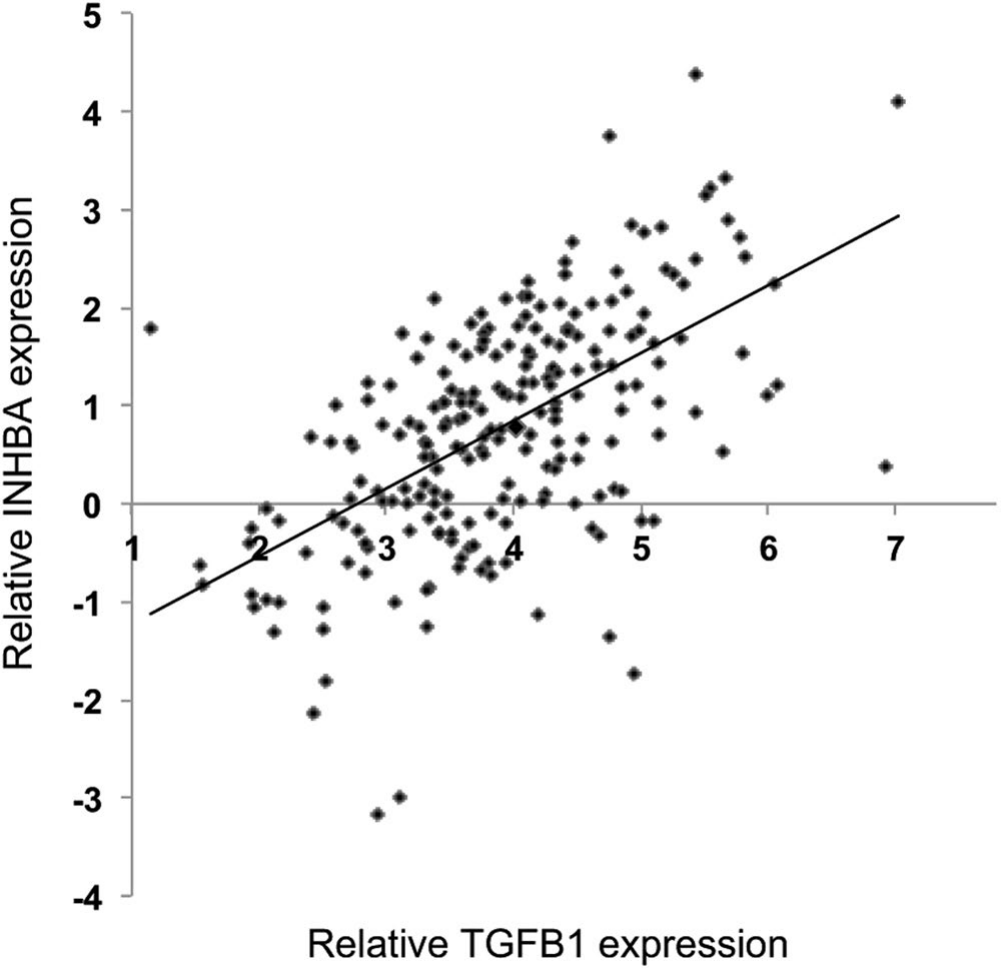
Activin and TGF-β ligand mRNA expression strongly correlate. Relative mRNA expression in the TCGA data set of the Activin A ligand subunit (INHBA) and TGF-β ligand (TGFB1) is shown after global normalization. Each data point represents an individual tumor (r = 0.6158).

We queried the connection of activin and TGF-β by assessing pathway member correlation in mRNA expression. Indeed, the expression of a substantial number of activin and TGF-β members correlated on the mRNA level (Table 1), most notably the activin ligand measurable subunit *INHBA* with the TGF-β ligand (*TGFB1*) (Fig. 3). In order to put the magnitude of the correlation into context, we performed a permutation test and correlated 100,000 random gene pairs (Supplementary Figure S3) revealing the correlation between activin and TGF-β ligand as in the top 1% of most strongly correlated genes in sporadic CRC (Table 1).

**Table 1:** Strong correlation of mRNA expression of activin and TGF-β pathway members.

Activin pathway	TGF-β pathway	rho	p-value	FDR
INHBA	TGFB3	0.666491996	<1E-22	<1E-22
INHBA	TGFB1	0.615817318	<1E-22	<1E-22
ACVR1	TGFBR1	0.601597339	<1E-22	<1E-22
ACVR2A	TGFBR1	0.574281616	<1E-22	<1E-22
ACVR1	TGFB2	0.50233367	<1E-22	<1E-22
INHBA	TGFB2	0.488423405	<1E-22	<1E-22
ACVR1	TGFB3	0.416297434	1.20E-12	7.78E-12
ACVR1	TGFBR2	0.345959021	6.60E-09	3.53E-08
INHBA	TGFBR1	0.335048949	2.04E-08	1.03E-07
ACVR2B	TGFBR2	0.3134617	1.68E-07	6.95E-07
ACVR2A	TGFBR2	0.300971359	5.30E-07	1.93E-06
ACVR1B	TGFBR2	0.294959388	9.05E-07	3.17E-06
ACVR2A	TGFB2	0.293090745	1.07E-06	3.59E-06

Activin and TGF-β ligands, receptor and downstream SMAD molecules were interrogated for correlation using Pearson’s correlation coefficient (rho). The p value of the correlation and the false discovery rate (FDR) are included.

These findings support the notion that activin and TGF-β pathways are closely connected in CRC. In addition, we found substantial *in silico* correlations through analysis in cohorts of other adenocarcinomas (esophageal adenocarcinoma TCGA provisional r = 0.58, p < 0.001, gastric adenocarcinoma TCGA^36^ r = 0.47, p < 0.01, lung adenocarcinoma TCGA^37^ r = 0.33, p < 0.05.) hinting towards a cross regulated TGF-β superfamily signaling network in additional solid tumors.

In summary, we here present *in vivo*, *in vitro* and *in silico* data all strongly supporting a close and functionally important connection between the activin and TGF-β pathways in advanced CRC.

## Discussion

Mortality from metastatic CRC remains high as prediction and treatment of metastasis are still ineffective. In addition, there is a growing number of patients under 40 years of age who present with advanced disease and did not meet age or risk requirements for screening^2^.

TGF-β, initially categorized as a tumor suppressor due to its growth suppressive properties, promotes metastatic CRC at later stages. TGF-β inhibitors are in early phase clinical trials, although results have been mixed^22^. We here present data suggesting that treatment options focusing on TGF-β alone may not be effective without considering the role of its superfamily member, activin. Activin and TGF-β signaling pathways have been deemed redundant due to shared downstream growth suppressive canonical signaling via SMAD4^8^. Our previously published studies support pro-metastatic actions of activin mechanistically distinct from TGF-β via separate effector mutagenic pathways^13, 32^. Here, we illustrate how activin and TGF-β are complexly intertwined and need to be interpreted as a unit to better target and predict metastatic CRC.

Here we show that simultaneously considering activin and TGF-β serum ligand levels is superior to assessing either ligand alone in identifying patients with advanced CRC and assessment of serum ligand levels is feasible as a clinical assay to guide treatment options. While baseline levels of activin appear to be low in the general populations (unpublished observations), clinical decisions based on elevated ligand serum levels have limitations in regards to measuring TGF-β superfamily signaling activation in CRC. Circulating levels of TGF-β and activin may be affected by other conditions, especially inflammatory conditions^38^. We determined here that a combined activin and TGF-β ligand expression score in the tumor was strongly predictive for shorter overall survival in our cohort, however, currently, there are no studies which simultaneously measures tumor and systemic ligand levels to determine if serum ligand levels reflect tumor ligand levels. Therefore, we plan to measure serum and tumor ligand expression in a prospective cohort including all CRC stages to fully evaluate their correlation and respective individual or combined predictive power. While we did not consider confounding factors such as lifestyle or mutational background in this initial study, it is possible and perhaps likely that our findings are influenced by confounders. Analyses of these clinical nuances will be important in effectively translating our observations into the clinical realm.

Follistatin, a naturally occurring activin antagonist which we utilized in our *in vitro* studies, was utilized by others to inhibit activin signaling and improve outcomes in *in vivo* models of inflammation^39, 40^. While follistatin is well tolerated, long term follistatin treatment for either *in vivo* models of CRC or patient care is challenging due to both its short half-life^41^ and considerable expense with repeat administration. Other approaches for inhibiting activin signaling are needed.

Our observation that activin inhibition through follistatin did not affect TGF-β-induced growth suppression suggests that activin inhibition may specifically inhibit pro-oncogenic TGF-β functions while preserving its growth suppressive functions. While the inhibition of activin signaling is understudied in comparison to the inhibition of TGF-β signaling, activin inhibition may have further clinical benefits including reversing both cancer associated cachexia^42, 43^ and anemia^44^. Additionally, activin inhibition has exhibited a favorable safety profile in a phase II study of cancer associated anemia^45^, which was unfortunately terminated early due to poor patient enrollment.

The co-occurrence of mutations in activin and TGF-β receptors we observed *in silico* strongly supports a functional connection between these pathways. This connection suggests substantial mutational pressure on the cancer cell to acquire a mutation in a TGF-β receptor if a mutation is present in an activin receptor, and vice versa. In our *in silico* analysis, mutations in receptors of both pathways were mutually exclusive with SMAD mutations, an established mechanism to escape TGF-β superfamily induced growth suppression^46^. This leads us to conclude that it is very likely that dual receptor mutations are an alternative route through which CRC can obviate the early anti-oncogenic effects of growth suppression by activin and TGF-β.

To our knowledge, this is the first report of a functional connection between activin and TGF-β signaling in CRC. In a mouse model of tumor cachexia, activin was shown to induce muscle wasting and inhibition of activin signaling reduced cachexia^42^. Others have reported activin to be downstream of TGF-β and necessary for its pro-metastatic function in breast adenocarcinoma cell lines^47^. These *in vitro* data and the data reported here suggest that assessing activin and TGF-β as a unit may be translatable to other solid tumors.

In summary, our study provides novel insight into the cross-regulation of TGF-β superfamily signaling in CRC and supports the assessment of activin signaling for both risk stratification and potential treatment of advanced CRC.

## Materials/Methods

### Reagents

Activin A and TGFβ1 were reconstituted in PBS plus 4 mM HCl according to the manufacturer’s instruction (both R&D, Minneapolis, MN). Follistatin 288 (R&D) was reconstituted in PBS.

### Cohorts

Serum samples from a subset of patients from the published Quasar colorectal cancer cohort^29^ were kindly provided by Dr. David Kerr as de-identified and coded. All samples were collected following informed consent and ethics approval for the study was given by the West Midlands Multi Research Ethics Committee at each collection site^29^. All methods were performed in accordance with the relevant guidelines and regulations of the local research ethics committee. The serum samples were drawn after surgery but prior to randomization into a chemotherapy arm versus a placebo arm. These samples consisted of serum samples from 120 stage II colon cancer patients. 60 patients without disease recurrence were matched by study arm, age, gender and BMI to 60 patients with disease recurrence. A commercial tumor micro-array of 40 CRC tumors, 10 metastasis and 10 control samples^30^ was employed to assay tumor activin and TGF-β ligand expression. The epidemiologic data of this cohort are listed in Table 2.

**Table 2:** Epidemiologic data of colorectal cancer TMA by Imgenex (San Diego, CA).

Stage	Stage I:	2 (5%)
	Stage II:	11 (27.5%)
	Stage III:	15 (37.5%)
	Stage IV:	12 (30%)
Metastasis	Yes:	13 (32.5%)
	No:	27 (68.5%)
Gender	Female:	12 (30%)
	Male:	28 (70%)
Age	< 65:	30 (75%)
	> 65:	10 (25%)
Activin/TGF-β staining intensity	High:	13 (32.5%)
	Intermediate/low:	27 (68.5%)
Survival (after 252 months of follow up)	Yes:	10 (25%)
	No:	23 (57.5%)
	Lost to follow up:	7 (17.5%)

Cohort data from commercial CRC tumor micro-array (TMA) including tumor stage, whether the subject had metastases, gender, age, length of survival, and activin and TGF-β combined staining intensity.

### Quantification of activin and TGF-β ligands

Activin and TGF-β from human patient serum samples and cell culture supernatants were measured utilizing activin A and TGFB1 Quantikine© ELISAs (both R&D Systems), respectively, following the manufacturer’s instructions. Cell culture supernatants were analyzed without prior freezing after 5 min centrifugation at 10,000 g, and human serum samples after storage at −80 °C. Activin and TGF-β ligand expression was assigned as score of “high” (3) if expression was higher than the 75th percentile in our cohort, “intermediate” (2) if levels were between the 25th and 75th percentile, and “low” (1) if levels were below the 25th percentile. Compound scores were calculated by adding activin and TGF-β scores. All samples were assessed in duplicates.

### Immunohistochemistry

Adjacent slides of a commercial TMA (Imgenex, San Diego, CA) with 40 CRC samples, 10 metastases and 10 adjacent normal samples were stained as previously reported^13, 25^ with antibodies against the Activin A subunit INHBA (Ansh lab LCC, Webster, TX) and TGFB1 (Abcam, Cambridge, United Kingdom). Slides were reviewed by a pathologist and scored blindly with an additional two individual investigators using a three point scoring system from 0 – weak staining to 2 - strong staining. A compound activin and TGF-β score was calculated by adding both scores.

### Cell culture

SW480 colon cancer cells (ATCC, Manassas, VA,) were maintained in DMEM, FET colon cancer cells (gift from Michael Brattain, University of Nebraska, Omaha, NE) and CCD18 fibroblasts (ATCC) cells were maintained in DMEM/F12 50:50 (both Corning Inc., Corning, NY) supplemented with 10% fetal bovine serum and penicillin (100 U/ml) as well as streptomycin (100 µg/ml) (Invitrogen, Carlsbad, CA). Cells were grown at 37 °C in a humidified incubator with 5% CO_2_. All cells were serum starved for 18 hours prior to treatment to approximate cell cycle synchronization.

Co-culture experiments were performed using trans-well plates (6-well) with 0.4 μm pore inserts. CCD18 fibroblasts were grown to confluency on the inserts and colon cancer epithelial cells (FET or SW480) on the plate surface. Cells were treated with either 10 ng/ml of TGF-β or activin and incubated for an additional 48 hours. Supernatant was collected and assayed for respective ligands by ELISA as described below.

All cell lines were validated for authenticity by 9 STR (short tandem repeat) profiling using CellCheck 9 Plus and tested negative for mycoplasma (both IDEXX, Columbia, MO).

### Transwell migration assay

Migration assays were performed as previously described^13, 32^. Briefly, transwell 12 well plates (8 µm pores, Corning) with fibronectin (Sigma, St. Louis, MO) were seeded with 5 × 10^5^ colon cancer cells per well. Cells were treated with either activin A (10 ng/ml) or TGF-β (10 ng/ml) or follistatin (100 ng/ml) for 30 minutes and TGF-β (10 ng/ml) was added. Cells were then allowed to migrate for 6 hours, stained, and imaged. Images from 5 microscopic fields at the center of each well were counted using ImageJ software^48^.

### Proliferation assay

Cells were seeded in 96 well plates, serum starved for 18 hours and treated with TGF-β at 10 ng/ml, follistatin 288 at 100 ng/ml, or a combination of both. Controls were treated with vehicle (PBS). After 48 hours, the wst-8-based Cell Counting Kit-8 (Dojindo Molecular Technologies, Inc., Rockville, MD) measuring metabolic activity as an approximation of cell proliferation was used following the manufacturer’s instructions as previously described^13^.

### *In silico* dataset

The Cancer Genome Atlas (TCGA) CRC dataset was accessed as described in its original publication^35^ which includes the clinical characteristics of this cohort. TCGA datasets for esophageal adenocarcinoma, gastric adenocarcinoma^36^, and lung adenocarcinoma^37^ were accessed via cbioportal.com^49, 50^.

### Statistical methods

Data are shown as mean + SEM if not explicitly stated otherwise. All *in vitro* experiments were reproduced at least twice. Statistical significance level of α = 0.05 was set before experiments. *In silico* analysis of the TCGA colorectal dataset was performed by the UIC biostatistical core facility. Mutual exclusivity and co-occurrence were tested using Fisher’s exact test. For correlation of expression, Pearson or Spearman’s correlation were used as applicable. For our permutation analysis, 100,000 random gene pairs from the TCGA colorectal dataset^35^ were correlated with each other. Two sided t-test with Welch’s correction and one sided ANalysis Of VAriance (ANOVA) with Dunett’s post-test was used for comparison of means in two or more than two groups with one control group respectively. For survival analysis, Log-rank (Mantel-Cox) test was used. All statistical analysis was performed using GraphPad Prism version 5.00 for Windows (GraphPad Software, San Diego, CA). All authors had access to the study data and reviewed and approved the final manuscript.

### Data Availability

All data generated or analyzed during this study are included in this published article (and its Supplementary Information files).

## Electronic supplementary material

Supplementary Material
