# Supplementary material for: Activin signaling is an essential component of the TGF-β induced pro-metastatic phenotype in colorectal cancer
Source: Sci Rep. 2017 Jul 17;7:5569. doi: 10.1038/s41598-017-05907-8 (PMC5514149; doi:10.1038/s41598-017-05907-8)
Supplement: Supplementary file 1 — Supplementary Material [file 41598_2017_5907_MOESM1_ESM.pdf]

Supplementary Material: **Activin signaling is an essential component of the TGF-β induced pro-metastatic phenotype in colorectal cancer.**

Authors: Jonas J. Staudacher[1], Jessica Bauer[1], Arundhati Jana[1], Jun Tian[1], Timothy Carroll[1], Georgina Mancinelli[1], Özkan Özden[1], Nancy Krett[1], Grace Guzman[2], David Kerr[3], Paul Grippo[1], and Barbara Jung[1]*

Figure S1.

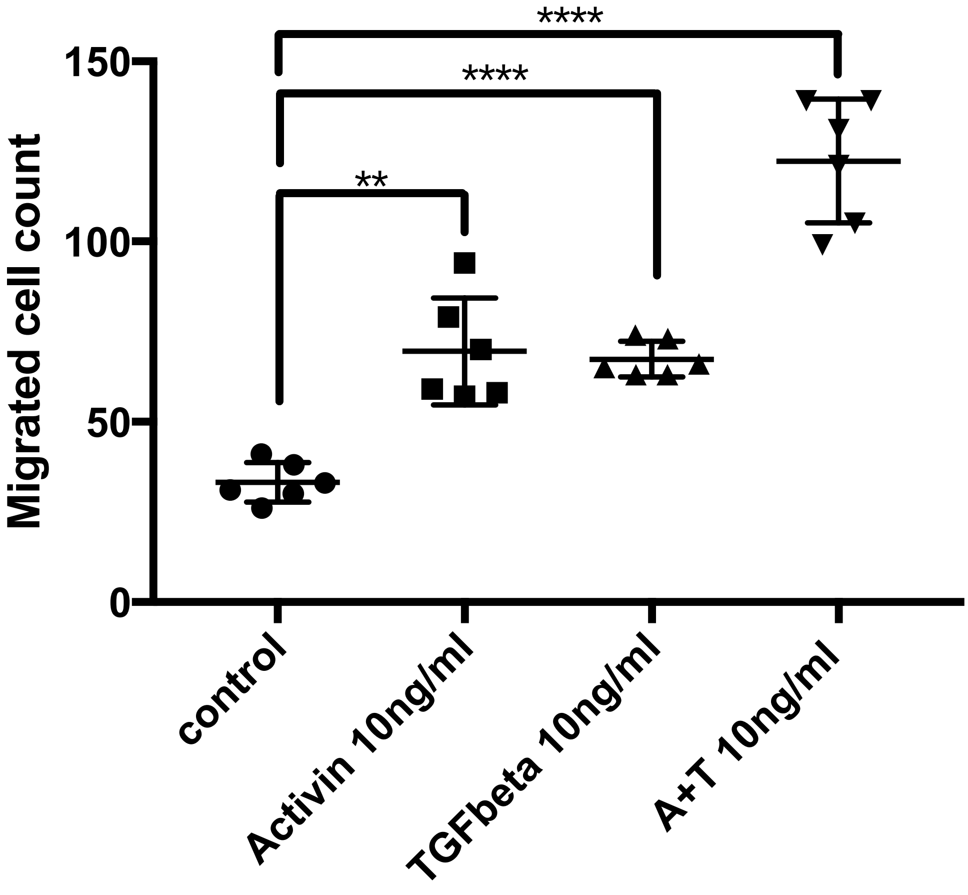

Supplementary Figure S1: Both activin and TGF-β induce colon cancer cell migration. Transwell migration assay of FET colon cancer cells treated with either activin (10 ng/ml) or TGF-β (10 ng/ml) or both (10 ng/ml of each ligand) as indicated. Cells were imaged and counted as described in Materials and Methods. Experiments were reproduced at least twice with a total n=6. **p=0.01 and ****p=0.0001.

Figure S2.

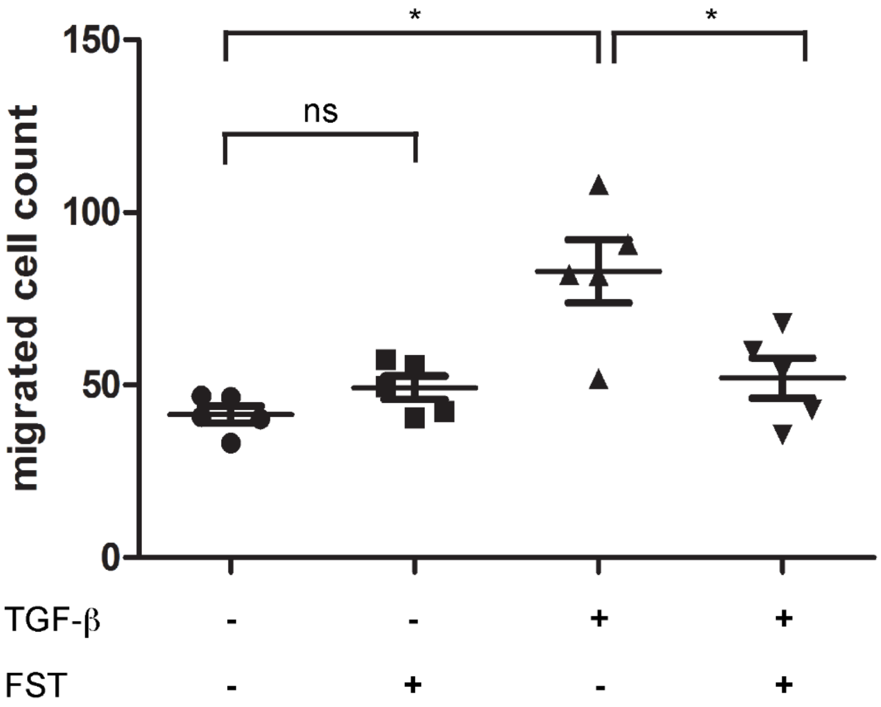

Supplementary Figure 2: TGF-β induced migration of SW480 colon cancer cells is abrogated by activin inhibition. Transwell migration assay of SW480 colon cancer cells treated with either follistatin (FST, 100 ng/ml) or TGF-β (10 ng/ml) or both as indicated. Cells were imaged and counted as described in Materials and Methods. Experiments were reproduced at least twice with a total n=5 and p=0.05.

Figure S3

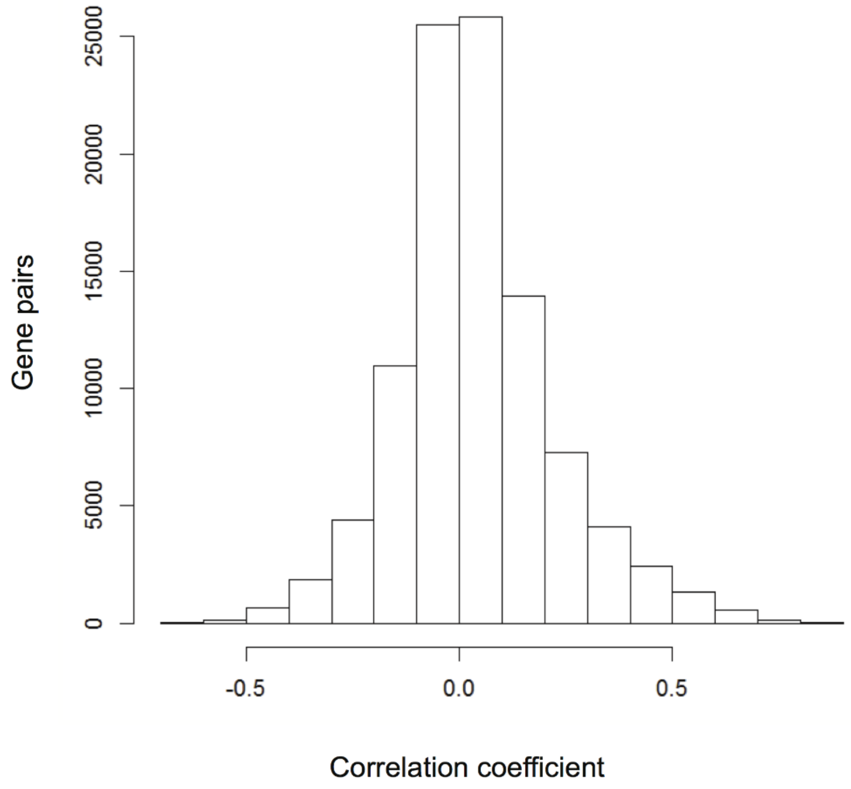

Supplementary Figure S3: Correlation coefficient of gene pairs. Correlation of gene pairs from the activin/TGF-β pathways are highly significant when compared to random gene pairs in CRC. 100,000 random gene pairs were correlated using Pearson's correlation coefficient. Frequency by magnitude of coefficient is shown indicating that the TGF-β and activin ligand correlation reported in Table 1 is in the top 1% of correlation in colorectal cancer.
